# 169. Positive blood culture after emergency department discharge of patients from a cancer center. Epidemiology, clinical features, and outcome

**DOI:** 10.1093/ofid/ofad500.242

**Published:** 2023-11-27

**Authors:** Pamela Alatorre Fernandez, Alex Cardona Ortiz, Beda Islas-Muñoz, Patricia Volkow-Fernández, Patricia Cornejo Juarez, Consuelo Velazquez Acosta

**Affiliations:** Instituto Nacional de Cancerología, Mexico City, Distrito Federal, Mexico; Instituto Nacional de Cancerología, Mexico City, Distrito Federal, Mexico; Instituto Nacional de Cancerologia, Mexico City, Distrito Federal, Mexico; Instituto Nacional de Cancerología, Mexico City, Distrito Federal, Mexico; Instituto Nacional de Cancerología, Mexico City, Distrito Federal, Mexico; Instituto Nacional de Cancerologia, Mexico City, Distrito Federal, Mexico

## Abstract

**Background:**

Cancer patients frequently go to the emergency room (ER) because of fever. Within the approach, blood cultures are taken, and they are often discharged if their clinical condition is stable. The objective of the study is to know the epidemiology, clinical characteristics, and outcome of patients with positive blood cultures after discharge from the emergency department.

**Methods:**

Between 2021 and 2022, 178 blood cultures taken in the ER were positive for infection. Of these, 93 cases (38%) corresponded to outpatients who were included. Demographic, clinical, and microbiological data were collected. A bivariate analysis comparing the cohort vs. patients that were admitted (controls) from the ER in the same period was done.

**Results:**

Most frequent oncological diagnosis was breast cancer (21.5%), lymphoma (18.3%), and cervical cancer (11.8%). Forty-nine (53%) had recently been diagnosed with cancer, 52 (55.9%) had received chemotherapy, and 15 (16.1%) had received antibiotics within the previous month. The most frequent reasons for consultation were febrile syndrome (28%), fever after CVC manipulation (20.4%), and fever with urinary symptoms (12%). Antibiotics were prescribed on discharge from the emergency department in 57 patients (61.3%), being adequate in 36.9% of the cases. The isolate was considered to be pathogenic in 84 cases, mainly due to Gram-negative bacilli (n=58, 69%). 72 (77.4%) attended reassessment (due to telephone contact or persistence of symptoms), and 56 (60.2%) were hospitalized. The most frequent bloodstream infections were secondary (n=45, 48.4%) and catheter-related (n=33, 35.5%). Three patients died, and only in one case was it secondary to an infection, compared to 23.5% in the admitted group. The bivariate analysis is reported in Table 1.

**Figure:**
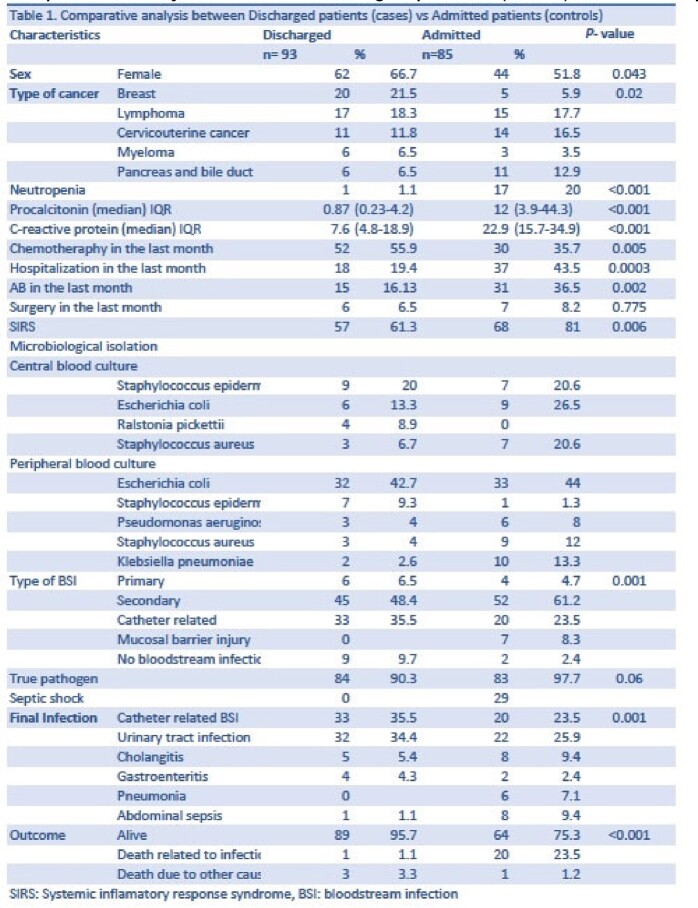

**Conclusion:**

Mortality in discharged patients was low (3%); Close monitoring of positive blood cultures, patient follow-up, and identification of risk factors for adverse outcomes, such as neutropenia, inflammatory response syndrome with high C-reactive protein or Procalcitonin, uncontrolled foci of infection, recent hospitalization, or antibiotic treatment should be considered for admission in this high risk population.

**Disclosures:**

**All Authors**: No reported disclosures

